# Maps of the Sri Lanka malaria situation preceding the tsunami and key aspects to be considered in the emergency phase and beyond

**DOI:** 10.1186/1475-2875-4-8

**Published:** 2005-01-27

**Authors:** Olivier JT Briët, Gawrie NL Galappaththy, Flemming Konradsen, Priyanie H Amerasinghe, Felix P Amerasinghe

**Affiliations:** 1 International Water Management Institute, P.O. Box 2075, Colombo, Sri Lanka; 2 Anti Malaria Campaign Head Office, Colombo, Sri Lanka; 3 Department of International Health, University of Copenhagen, Denmark

## Abstract

**Background:**

Following the tsunami, a detailed overview of the area specific transmission levels is essential in assessing the risk of malaria in Sri Lanka. Recent information on vector insecticide resistance, parasite drug resistance, and insights into the national policy for malaria diagnosis and treatment are important in assisting national and international agencies in their control efforts.

**Methods:**

Monthly records over the period January 1995 – October 2004 of confirmed malaria cases were used to perform an analysis of malaria distribution at district spatial resolution. Also, a focused review of published reports and routinely collected information was performed.

**Results:**

The incidence of malaria was only 1 case per thousand population in the 10 months leading up to the disaster, in the districts with the highest transmission.

**Conclusion:**

Although relocated people may be more exposed to mosquito bites, and their capacity to handle diseases affected, the environmental changes caused by the tsunami are unlikely to enhance breeding of the principal vector, and, given the present low parasite reservoir, the likelihood of a malaria outbreak is low. However, close monitoring of the situation is necessary, especially as December – February is normally the peak transmission season. Despite some losses, the Sri Lanka public health system is capable of dealing with the possible threat of a malaria outbreak after the tsunami. The influx of foreign medical assistance, drugs, and insecticides may interfere with malaria surveillance, and the long term malaria control strategy of Sri Lanka, if not in accordance with government policy.

## Background

After the tsunami hit Sri Lanka on 26 December 2004, news reports and public health agencies warned against the possibilities of an increase of vector borne diseases, in particular malaria and dengue. Immediately after the disaster, an estimated 860,000 people were displaced and more than 820 emergency camps established throughout the affected areas [1]. By 14 January, approximately 440,000 people were still sheltered in approximately 460 emergency camps [2]. Maps of the tsunami affected area, are presented elsewhere [3].

Malaria in Sri Lanka is of a highly unstable nature and has historically fluctuated greatly over the years and with significant seasonal differences. Sixty-five to eighty percent of the malaria cases are caused by *Plasmodium vivax *and the remainder by *Plasmodium falciparum *[4]. Recently, an overview of the spatial and temporal distribution of malaria in Sri Lanka over the period 1995 – 2002 was published in this journal [5]. The present publication aims at providing an update on the recent malaria situation, to October 2004 inclusive, and to discuss factors of relevance which may help in assessing the potential of the tsunami and ensuing events for exacerbating the malaria situation.

## Methods

**Figure 1:**
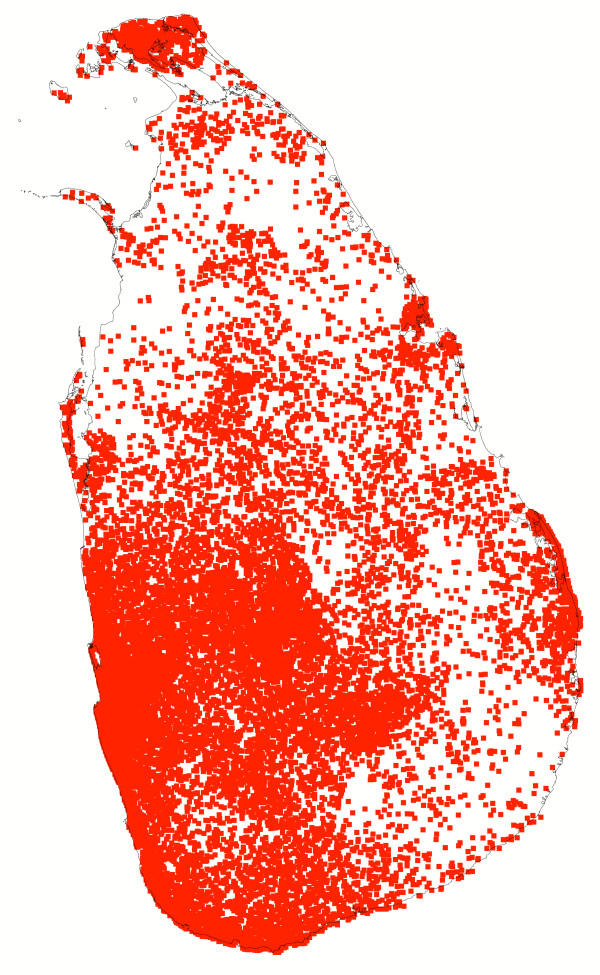
**Population. **Map of population by divisional secretariat division in Sri Lanka estimated for mid December 2004. One dot represents 1,000 people. Sources: Department of Census and Statistics , North East Provincial Council  and UNCHR .

Malaria maps were based on monthly records over the period January 2004 – October 2004 (the most recent month for which data recording was complete at the time of writing) of microscopically confirmed malaria parasite positive blood smear readings, at district spatial resolution. These were collected by the Anti Malaria Campaign (AMC) Directorate of the Ministry of Health from aggregated disease records reported by governmental hospitals and mobile clinics. Additionally, in the temporal analysis, monthly data by district for the period 2001 – 2002, and data by sub district for 1995 – 2000 as described by Briët *et al*. [5] were used. The quality of routinely collected information on malaria is described elsewhere [5]. As denominator for the incidence calculations, population estimates for 2001 and beyond were made by exponential interpolation (and extrapolation to December 2004) (Figure 1) as follows. For the districts Mannar, Vavuniya, Trincomalee and Batticaloa, that were not or incompletely enumerated in the 2001 census because of limited access of the government to these conflict affected areas, the 2001 mid-year population was taken from data posted by the North East Provincial Council [6]. For all other districts, the 2001 mid-year population was taken from data posted by the Department of Census and Statistics [7]. The natural annual (mid-2001 to mid-2002 and mid-2002 to mid-2003) population growth rates for Jaffna, Kilinochchi, Mullaitivu, Mannar, Vavuniya, Trincomalee and Batticaloa were taken as the average annual growth rates of all the other districts, calculated from mid year population statistics estimated by the Department of Census and Statistics. For all other districts, these growth rates were calculated for each district separately. For mid 2003 to mid 2004 and beyond, the growth rates for mid-2002 to mid-2003 were used. Further, the number of internally displaced persons (IDPs) was taken into account [8]. For each month and for each district, the net number of immigrants was calculated as the total number of IDPs moved to or within a district since 2001, minus the number of IDPs moved from or within that district. This net number of immigrants was then distributed over the months proportionately to the monthly statistics of IDPs moved to or within a district. Additionally, the number of monthly immigrants from India was taken into account.

A focused review of literature has been performed, identifying crucial information for the outbreak preparedness and control during the emergency phase. The intent was not to present a complete review of malaria in Sri Lanka but to provide information useful for an assessment of the current situation. A general review of malaria in Sri Lanka can be found in Konradsen, Amerasinghe *et al*. [4].

## Results and discussion

### Present malaria situation and parasite reservoir

**Figure 2:**
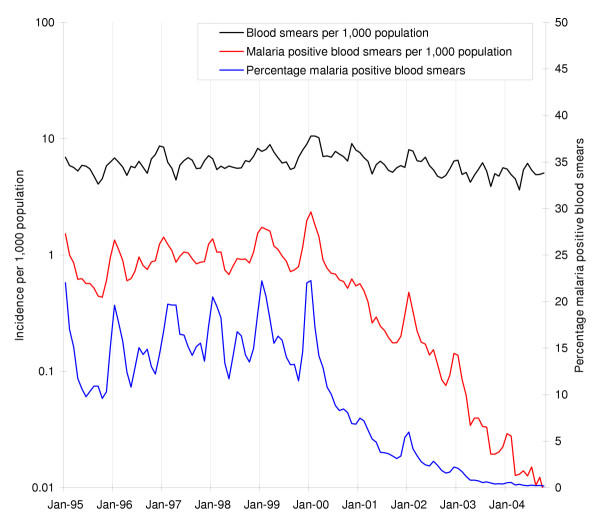
**Monthly parasite and blood smear examination incidence patterns. **Monthly parasite incidence patterns of *P. falciparum *and *P. vivax *malaria combined per 1000 population (red line on logarithmic scale), blood smears examined per 1000 population (black line on logarithmic scale), and percentage of blood smears positive for malaria (blue line) from January 1995 to October 2004 in Sri Lanka.

**Figure 3:**
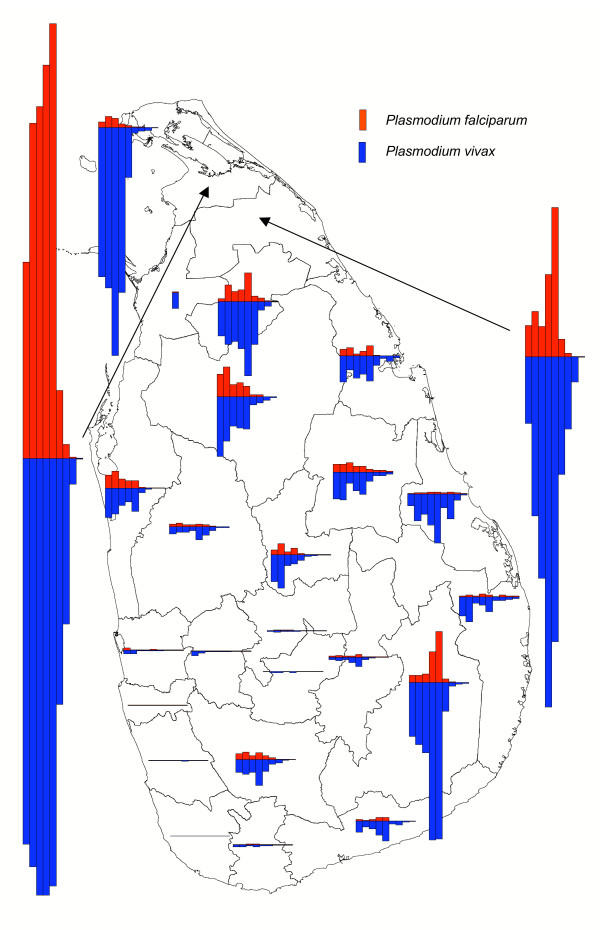
**Trends of parasite incidence. **Trends of parasite incidence of *P. falciparum *(red bars) and *P. vivax *(blue bars) malaria over the years November 1995 – October 1996 (bar on far left) to November 2003 – October 2004 (bar on far right), at district resolution. The height of the bars in the legend represents an annual parasite incidence of 10 cases per 1000 persons.

**Figure 4:**
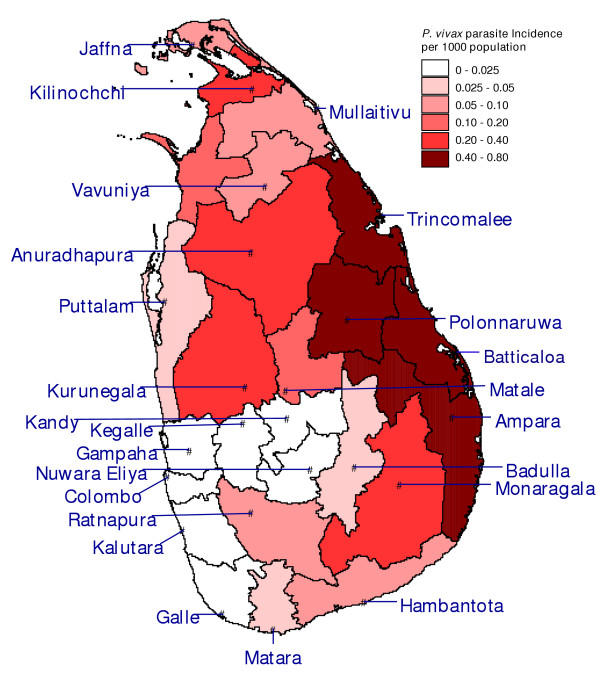
**Parasite incidence of *Plasmodium vivax. ***Map of the districts of Sri Lanka with *P. vivax *malaria cases per 1000 population over the period January – October 2004.

**Figure 5:**
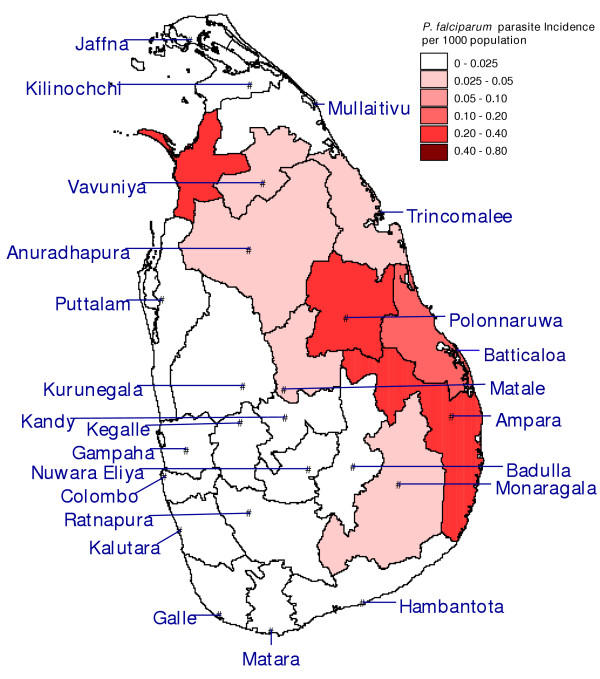
**Parasite incidence of *Plasmodium falciparum. ***Map of the districts of Sri Lanka with *P. falciparum *malaria cases and mixed infections of both *P. vivax *and *P. falciparum *per 1000 population over the period January – October 2004.

The country-wide malaria incidence increased from January 1996 to January 2000, with the typical seasonality of high peaks around January and lower peaks around June – July, but it has decreased dramatically since January 2000 (Figure 2). Figure 3 shows that the recent decrease in the overall malaria incidence in the country is predominantly due to a decrease in incidence in the districts of Vavuniya and Kilinochchi in the north. The decrease was least in the district of Ampara, making it the most malarious district during January to October 2004 (Figures 4 and 5). Although districts on the east coast which were badly affected by the tsunami had been relatively malarious in 2004 as compared to the rest of the country, the maximum of around 1 case per 1000 people over a 10 month period in these districts is remarkably low. The total number of malaria cases in 2003 was 10,510, the lowest since the resurgence of malaria in 1968 when the eradication campaign failed [9]. The year 2004 promises to be three times lower with only 3,037 cases recorded up to October, as opposed to 9,682 cases recorded during January – October 2003. The low incidence is not related to a decline in collection effort, which has decreased only marginally (Figure 2). At the time of writing, malaria incidence information for the months of November and December was still incomplete. In November 2004, without the figures for the non endemic districts Gampaha and Kalutara, and data from a few medical institutions in Mannar and Mullaitivu missing, thus far only 230 cases were recorded. In the malaria endemic districts, December, January and February are normally the months with the highest malaria incidence [5], so a rise in case numbers may normally be expected. However, neither the district authorities nor the Epidemiology Unit of the Ministry of Health have reported any malaria cases from the affected areas for 30 December 2004 – 13 January 2005, based on the spot checks performed and the review of available health information [10]. Asymptomatic infections of *P. falciparum *and *P. vivax *and dormant stages of *P. vivax *normally provide the parasite reservoir for bridging periods of low seasonal transmission (with unsuitable conditions for mosquito vectors). Under the present policy of administering primaquine in addition to chloroquine (see section on diagnosis and treatment), the reservoir of dormant stages of *P. vivax *will be low and this will delay a possible outbreak. It must be emphasized that the low level of malaria transmission in the recent past does not guarantee that localized or even island wide epidemics will not occur. In the past, even after periods of very low levels of malaria transmission, outbreaks have occurred, often due to constraints placed on the public health system, by unusual rainfall patterns or by yet unexplained factors.

### Capacity of health care services and disease surveillance

An important factor to consider in the current situation is the capacity of the existing health care service. Following the tsunami the Sri Lanka Ministry of Health reported 22 hospitals and nine administrative buildings damaged or completely destroyed, mostly in Ampara and Trincomalee districts [11]. It has been reported that at least 40 doctors and hundreds of other medical staff have died as a consequence of the tsunami and a much higher number injured or in other ways affected by the disaster [12]. However, both the central government departments and organizations in the field report sufficient medical staff. Even in the conflict affected areas in the north and east, the AMC has been able to monitor malaria and react timely with control measures to outbreaks since the peace process started in 2002. Also, the AMC has long standing experience with mobile clinics for malaria detection and treatment in remote areas. Lack of co-ordination among the many government departments, international aid agencies, non-governmental organizations and private individuals involved in the first phase of the emergency continues to be an important issue weeks into the disaster. According to the Ministry of Health media reports, more than 600 foreign doctors are now working in the affected areas, but few, if any, are registered with the Sri Lanka Medical Council or other relevant authorities [13]. With doctors from many countries, language barriers are also a perceived problem.

In some places, central stocks of medical supplies were destroyed, including the Regional Medical Supply Division in the Ampara District. However, sufficient drugs have been imported during the days and weeks following the disaster. The World Health Organization has drawn up plans for antimalarials, insecticides and spray equipment to be made available on request. Although the increased capacity at the district and provincial levels has improved the co-ordination, a risk remains that local needs for health care are not adequately covered in spite of the availability of significant resources. In some parts of the island, especially areas in the east, affected both by the destruction caused by the tsunami and by exceptionally heavy rainfall in the weeks following, distribution of drugs has been problematic and this has left certain communities vulnerable.

Whereas the overall capacity to provide treatment and routine malaria control activities, in general, has not been severely hampered, the routine health information system will have been constrained by the large number of autonomous health camps set up, and their lack of integration with the established surveillance system. It is essential to establish a system for monitoring malaria in the affected areas. Many people are moving back to their old place of residence trying to rebuild livelihoods and it will be essential for the public health authorities to keep contact with these communities to prevent an increase in malaria going unnoticed.

### Diagnosis, treatment and drug resistance

In Sri Lanka, microscopy on blood smears or use of rapid diagnostic test kits have been the standard diagnostic procedure, and precedes the prescription of drugs to the patient. In the current situation, with the many small health clinics established within emergency camps, it is likely that the use of rapid diagnostic kits would be the more feasible means of confirmation. The first line drugs recommended for malaria treatment in Sri Lanka is still a chloroquine and primaquine (PQ) combination for cases of *P. vivax *as well as *P. falciparum *infection. Primaquine is not administered to children below one year, and those with known G-6PD enzyme deficiency, and for pregnant mothers.

So far, there have been no reports of chloroquine-resistant *P. vivax *infections in Sri Lanka. The first chloroquine-resistant *P. falciparum *case was reported in 1984 [14]. Up to 62% *in vivo *chloroquine resistance has been recorded in malarious areas [5,15-17]. For chloroquine resistant cases of *P. falciparum *the government recommended drug is sulphadoxine-pyrimethamine (SP). However, SP is not recommended for the last trimester of pregnancy, first six weeks of lactation and for children below two months of age. The first SP-resistant case of *P. falciparum *was reported in 1992 in Polonnaruwa district. Up to 1999, five to six cases have been reported by the AMC. More recently (January – June 2002), SP resistant *P. falciparum *has been documented in the Northern Province [17]. For SP resistant cases quinine is recommended, but only as an in-patient treatment.

In the current emergency situation, with many (foreign) doctors working autonomously, the diagnosis and treatment practices may depart from the established government guidelines and new antimalarials are also likely to be brought in. Moreover, the current practice of restricting SP to government hospitals will be difficult to enforce. Similarly, introduction of low quality and obsolete drugs will be difficult to counter at community level at the current stage of supervisory capacity and co-ordination level. Drugs have been reported stolen from warehouses, allegedly finding their way to private trade establishments [18]. Overall, it is crucial that the development of drug resistance is monitored closely and inappropriate drugs are actively phased out of the market to avoid later complications in case management.

### Environmental changes and vector breeding

The seawater brought inland by the tsunami has mixed with monsoon rainwater to form puddles of varying salinity. Also, thousands of muddy surface water puddles have been created as a result of destruction and rehabilitation activities that are already underway. The brackish puddles are expected to favour the breeding of *Anopheles subpictus *sibling species B, which is a well-known coastal breeding species in Sri Lanka. However, it has not been directly incriminated as a field vector in Sri Lanka, despite its susceptibility to *P. falciparum *[19]. Nevertheless, Abhayawardana et al. [20] found peak malaria transmission in coastal areas of Puttalam in the presence of *An. subpictus *sibling species B and the complete absence of *Anopheles culicifacies *(the main malaria vector in Sri Lanka), and suggested that this *An. subpictus *sibling may have a role in transmission. It is noteworthy, that freshwater *An. subpictus *(which is now known to consist of a mixture of species A, C and D), which breeds in muddy rain fed puddles, has been consistently incriminated in malaria transmission in many inland areas of Sri Lanka [4]. Another species that is likely to breed prolifically in muddy rain-fed pools is *Anopheles vagus*. This species has been linked as a vector responsible for a malaria outbreak in southern Sri Lanka [4,21]. On present evidence, neither *An. subpictus *nor *An. vagus*, are likely to cause major malaria epidemics but could, at high density, be responsible for focal outbreaks that need quick action. Thus, it is important that an entomological monitoring programme be set up in the period leading up to and during the south west monsoon that is expected during May to June 2005 in the tsunami affected western and southern Sri Lanka. It should be noted that the infamous Asian brackish water breeding malaria vector *Anopheles sundaicus*, which is a threat in the tsunami-affected areas in Indonesia, Myanmar, and the Andaman and Nicobar islands [22], does not occur in Sri Lanka.

The main vector in Sri Lanka is *An. culicifacies *type E [23,24], which breeds mainly in pools formed in river and stream beds, and therefore, its density is mostly dependent on temporal and spatial variations in rainfall and river flow. *Anopheles culicifacies *also breeds in abandoned gem mining pits, agricultural wells and to a lesser extent in pools in agricultural water reservoirs [4]. It is unlikely that the rubble constituting a major part of the landscape in the affected areas creates breeding opportunities for *An. culicifacies*, unless it blocks waterways and creates pooling. Post-tsunami development activities may revive banned sand mining practices in rivers. If this happens, clear water pools created by these sand mining activities may serve as breeding sites for *An. culicifacies *[4]. Overall, it is very unlikely that the principal vector of malaria in Sri Lanka will breed prolifically in brackish water habitats or other habitats that may be created during the post tsunami reconstruction phase. Similarly, the principal dengue vector in Sri Lanka, *Aedes aegypti*, does not breed in saline water [25]. However, it may find plenty of rainwater-filled containers amidst the rubble created by the disaster for it to breed.

### Vector control strategies and insecticide resistance

The Colombo based Head Office of the AMC gives the overall guidelines for island wide vector control, while each province works out a plan for control activities based on the distribution and level of malaria transmission. Several malaria vector control interventions are currently employed within the country. In all districts, residual insecticide spray activities are focused on areas where malaria transmission has been established by confirmed malaria cases. The control of anopheline larvae using mostly chemicals focuses on sites close to human habitation. Small-scale application of larvivorous fish and environmental modifications are also carried out. Since 1997, mosquito nets, which are biannually treated with insecticide, are distributed free of charge in malarious areas. During the last two years, the main control effort has been through these nets. Since January 2004, 80,000 nets with long lasting insecticide have been distributed. Also, nets are available for purchase from outlets in most parts of the country.

Studies in Sri Lanka over the 1990s on *An. culicifacies *and a range of potential secondary vectors such as *An. subpictus *and *An. vagus *have shown high level of resistance to either organochlorines, organophosphates or to both groups of insecticides [4,26-28]. DDT and Malathion are no longer recommended since *An. culicifacies *and *An. subpictus *has been found resistant. Currently, synthetic pyrethroids such as Cyfluthrin, Deltamethrin, Etofenprox, and Lambda-cyhalothrin are being used in the country. At present, Fenitrothion is the only organophosphate used for vector control. A study conducted by Abhayawardana from 1990 to 1992  on *An. subpictus *found 68% and 54% susceptibility to Malathion and Fenitrothion, respectively, for inland species (sibling species A), whereas for coastal species (primarily sibling species B) it was 100%. However, the latter was found resistant to permethrin [20]. From several districts it was reported that, as a result of the tsunami, organisations have brought in insecticides not normally used or no longer recommended for vector control in Sri Lanka (P. Amerasinghe, personal communication). Vector resistance, in the light of the introduction of new insecticides, needs to be monitored and if necessary action should be taken.

### Exposure of the affected community

The majority of the people initially affected by the disaster are still living in emergency camps or in other places close to the coast. At the time of writing, to the best of our knowledge, relatively few people have moved from areas of low or no malaria transmission to areas of high transmission. However, during the next phases, when people may be resettled in semi-permanent and later in permanent housing, communities may be relocated from areas where they have had no malaria experience to malarious areas. In these situations, the communities' capacity to cope with malaria infection will be low.

Despite distribution of nets to many camps, and intensified vector control in some areas, people in the emergency camps (schools, temples, mosques, etc.) and those returning to damaged houses are more exposed to mosquito bites than in pre-disaster housing, due to the open nature of the shelter. Additionally, most families will have lost mosquito nets or other means to protect against mosquito bites. It is more difficult to assess the protective effect of tents that have been set up in most of the semi-permanent camps established. The location of semi permanent and permanent settlements may have a significant effect on the risk of infection. Epidemiological studies from other parts of Sri Lanka have shown that people living within 750 m of a stream with *An. culicifacies *breeding, were at significantly higher risk for malaria than people living further away [29].

## Conclusions

This paper provides maps of both *P. vivax *and *P. falciparum *malaria incidence distribution on the island of Sri Lanka at district resolution in the 10 months preceding the tsunami, and an analysis of monthly malaria incidence in the country since January 1995. The malaria incidence was historically low, which implies a limited parasite reservoir in the human population. In spite of the fact that the months of December - February are normally the peak period for transmission, given the transmission level in the months leading up to the disaster, the risk of a large-scale outbreak seems to be limited. However, the low transmission levels over the past years may also have made people less alert to possible outbreaks, and the population would have less protective immunity towards the disease. The environmental changes resulting from the tsunami do not create particular opportunities for breeding of the principal malaria vector *An. culicifacies *but potential does exist for less important species such as *An. subpictus *and *An. vagus*. People living in emergency camps or returning to pre-disaster areas of residence are at higher risk of mosquito bites than normal. In spite of the emergency, the capacity of the public health authorities to perform malaria preventive and curative interventions remains high and essential supplies and staff capacity is not a problem. However, co-ordination of assistance and maintaining a strong surveillance system remain significant areas of concern. Increased attention to the establishment of a monitoring system including both parasitological and entomological parameters is recommended. Likewise, the large inflow of donated drugs and insecticides outside government control will potentially have long term implications on malaria control and case management, and especially the quality of administered drugs and the development of drug resistance requires careful monitoring.

## Authors' contributions

GNLG collected the malaria data. OJTB checked the data, calculated incidence, made the maps. FK, FPA and PHA performed the focused literature review. All authors helped write, read and approved the final manuscript.
